# Supplementary material for: Mammalian SIRT6 Represses Invasive Cancer Cell Phenotypes through ATP Citrate Lyase (ACLY)-Dependent Histone Acetylation
Source: Genes (Basel). 2021 Sep 21;12(9):1460. doi: 10.3390/genes12091460 (PMC8466468; doi:10.3390/genes12091460)
Supplement: Supplementary file 1 [file genes-12-01460-s001.zip › genes-1340825-supplementary.pdf]

Supplementary Figure S1

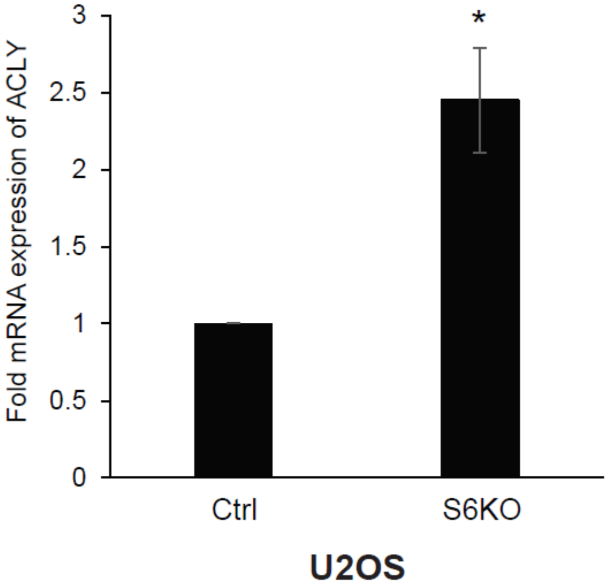

Supplementary Figure S1. ACLY mRNA levels in SIRT6-deficient U2OS cells. Increased expression of ACLY mRNA in S6KO compared to control U2OS cells, normalized to GAPDH (Mean ± SEM of 3 independent experiments, * $p < 0.05$, two-tailed Student's t-test).

Supplementary Figure S2

A

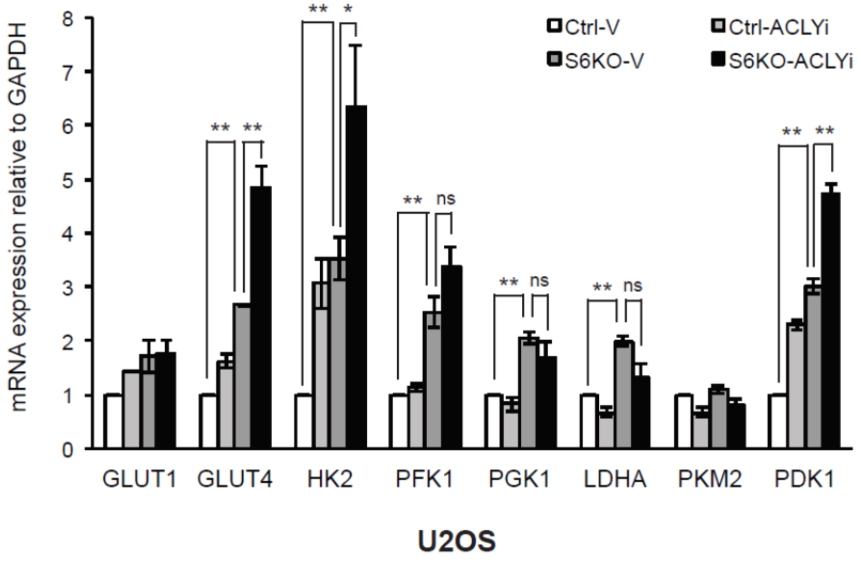

B

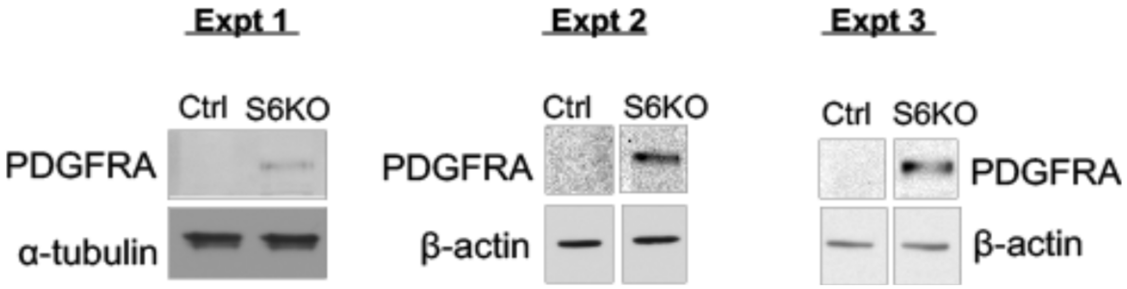

Supplementary Figure S2. Impact of SIRT6-deficiency and ACLY inhibition on expression of major glycolytic genes and PDGFRA in U2OS cells. **A.** mRNA levels of major glycolytic genes in control and SIRT6 KO U2OS cells treated with 50 uM ACLY inhibitor for 24 hours (Mean ± SEM of 3 independent experiments, * $p < 0.05$, ** $p < 0.01$, ns: not significant, two-tailed Student's t-test). **B.** Western blots showing increased PDGFRA protein in SIRT6 knockout (S6KO) U2OS cells. Three independent experiments are shown. Tubulin or beta-actin are shown as loading controls. In experiments 2 and 3, the lanes are cropped to remove irrelevant lanes between them, and the control and S6KO lanes show identical exposures.

Supplementary Figure S3

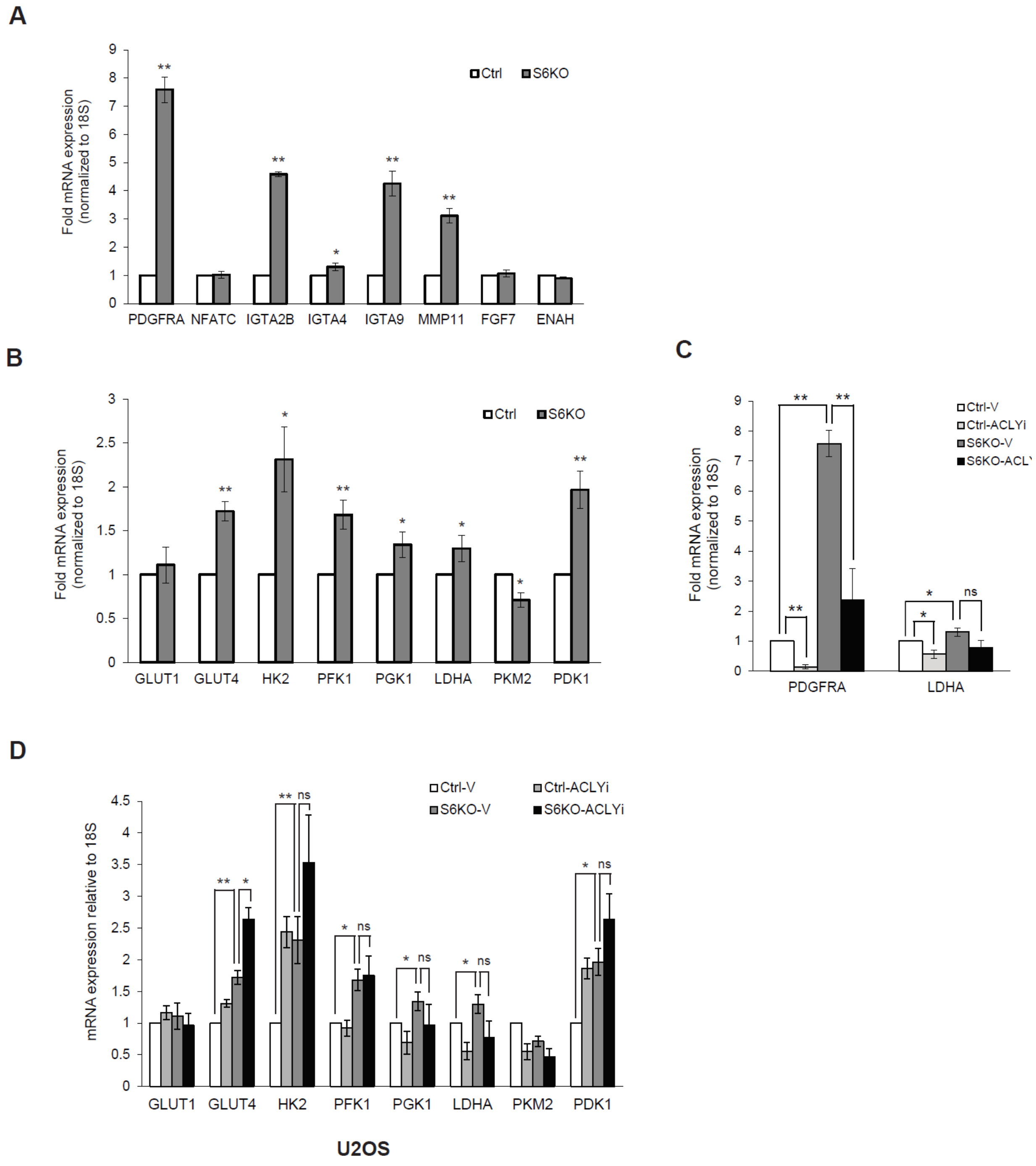

Supplementary Figure S3. qPCR data analysis of mRNA levels normalized to 18S rRNA in U2OS cells. **A.** Expression of acetyl-CoA responsive genes in control and SIRT6 KO U2OS cells normalized to 18S rRNA (Mean ± SEM of 3 independent experiments, * $p < 0.05$, ** $p < 0.01$, two-tailed Student's t-test). **B.** Expression of glycolytic genes in control and SIRT6 KO cells normalized to 18S rRNA (Mean ± SEM of 3 independent experiments, * $p < 0.05$, ** p <

0.01, one-tailed Student's t-test). **C.** Increased expression of PDGFRA but not LDHA in SIRT6 KO cells is attenuated by ACLY inhibitor (ACLYi, 50 uM; 24 hours). Data are normalized to 18S rRNA and show Mean ± SEM of 3 independent experiments, * $p < 0.05$, ** $p < 0.01$, ns: not significant, one-tailed Student's t-test). **D.** Expression of glycolytic genes in control and SIRT6 KO cells treated with 50 uM ACLY inhibitor for 24 hours, normalized to 18S rRNA (Mean ± SEM of 3 independent experiments, * $p < 0.05$, ** $p < 0.01$, ns: not significant, one-tailed Student's t-test).

Supplementary Table S1

**Table S1 Primers used in QPCR analysis**	
**mRNA Expression**	**Primer sequence**
GLUT1	Forward: 5'- TATCGTCAACACGGCCTTCACTGT-3'<br>Reverse: 5'- CACAAAGCCAAAGATGGCCACGAT-3'
GLUT4	Forward: 5'- CAACAGATAGGCTCCGAAGATG-3'<br>Reverse: 5'- CCAAGCCACTGAGAGATGATAC-3'
LDHA	Forward: 5'- TGGTCCAGCGTAACGTGAACATCT-3'<br>Reverse: 5'- TTGCAACCGCTTCCAATAACACGG-3'
HK2	Forward: 5'- CTGCAGCGCATCAAGGAGAACAAA-3'<br>Reverse: 5'- ACGGTCTTATGTAGACGCTTGGCA-3'
PGK1	Forward: 5'- TCACTCGGGCTAAGCAGATTGTGT-3'<br>Reverse: 5'- CGTGTTCCATTTGGCACAGCAAGT-3'
PDK1	Forward: 5'- TCATGTCACGCTGGGTAATGAGGA-3'<br>Reverse: 5'- AACACGAGGTCTTGGTGCAGTTGA-3'
PFK1	Forward: 5'- GTGGCTCTAAACTTGGGACTAAA-3'<br>Reverse: 5'- GTTGGAGACTGTAGCAGGAATG-3'
PKM2	Forward: 5'- ATTATTTGAGGAACTCCGCCGCCT-3'<br>Reverse: 5'- CATTCATGGCAAAGTTCACCCGGA-3'
